# Ceramic-on-ceramic articulation in press-fit total hip arthroplasty as a potential reason for early failure, what about the survivors: a ten year follow-up

**DOI:** 10.1007/s00264-020-04895-1

**Published:** 2021-01-18

**Authors:** J. van Loon, A. M. J. S. Vervest, H. M. van der Vis, I. N. Sierevelt, D. C. Baas, K. T. M. Opdam, G. M. M. J. Kerkhoffs, D. Haverkamp

**Affiliations:** 1 Department of Orthopedic Surgery, Xpert Clinics/SCORE (Specialized Center of Orthopedic Research and Education), Laarderhoogtweg 12, 1101EA Amsterdam, The Netherlands; 2 Department of Orthopaedic Surgery, Amsterdam University Medical Centers, location Academic Medical Center, Meibergdreef 15, 1105 AZ Amsterdam, The Netherlands; 3 Department of Orthopaedic Surgery, Tergooi, Van Riebeeckweg 212, 1213 XZ Hilversum, The Netherlands; 4 Department of Orthopaedic Surgery, Spaarne Gasthuis, Spaarnepoort 1, 2134 TM Hoofddorp, The Netherlands

**Keywords:** Aseptic loosening, Early failure, Total hip arthroplasty, Ceramic-on-ceramic, Primary stability

## Abstract

**Purpose:**

In press-fit total hip arthroplasty (THA), primary stability is needed to avoid micromotion and hereby aseptic loosening, the main reason for early revision. High aseptic loosening revision rates of the seleXys TH+ cup (Mathys Medical) with Ceramys ceramic-on-ceramic (CoC) bearing are seen in literature. Since CoC is presumed to overcome long-term wear-related revisions, the reason for early failure of this cup is important to clarify. The aim is to investigate its ten year outcomes and differentiate between potential causes and identify risk factors for aseptic loosening.

**Methods:**

Retrospective screening of a prospectively documented series of 315 THAs was performed. Primary outcome was cumulative incidence of cup revision due to aseptic loosening. Secondary outcomes were component revision and reoperation. Additionally, potential predictive factors for aseptic loosening were evaluated.

**Results:**

At the median follow-up of 9.7 years [IQR 4.4; 10.3], 48 TH+ (15.2%) were revised due to aseptic loosening. Competing risk analysis showed a ten year cumulative incidence of cup revision due to aseptic loosening of 15.6% (95% CI 12.0–20.2). Stabilization of early revision rates was observed, following a high rate of respectively 81.3% (*n* = 39) and 95.8% (*n* = 46) within the first two and three years. No significant predictive factors for aseptic loosening were found.

**Conclusion:**

The ten year results of seleXys TH+ cup with Ceramys CoC bearing showed an unacceptable high aseptic loosening rate, which stabilized over time after a high early failure incidence. This could be attributed to a problem with osseointegration during the transition of primary to definitive stability.

## Introduction

During press-fit total hip arthroplasty (THA), the initial primary stability of the uncemented acetabular cup during implantation is the most important factor for survival of the implant [1, 2]. Sufficient primary stability, avoiding micromotion, is needed to form fibrous or fibrocartilaginous tissue, and subsequently bony tissue, which causes osseointegration [3]. Micromotion jeopardizes osseointegration and therefore definitive secondary stability, which can cause aseptic loosening of the implant, one of the main reasons for early revision in THA [4–6]. Focusing on the long term, aseptic loosening caused by wear-induced osteolysis is regarded as the main limitation of prosthesis survival [7, 8]. To overcome both of these problems, the search for the perfect implant still continues. Ceramic-on-ceramic (CoC) is one of the options to overcome wear and late revisions. This hard-on-hard bearing shows wear rates of 5 μm/year compared with 50 μm/year in ceramic-on-polyethylene (CoPE) bearing during 20 years [9].

The press-fit seleXys TH+ cup (Mathys Medical) with a flattened pole and thick wall to prevent deformation was specially designed for both ceramic and polyethylene and metal-on-metal inlays. The titanium alloy cup has a surface of a corundum-blasted microstructure for optimal roughness (Ra 6–12 μm) with an equatorial macrostructure with tetrahedrons (TH+) with a height of 0.65 ± 0.1 mm. The initial fixation results from a 2-mm oversizing of the cup compared with the last used reamer size. Short-term results of this implant were previously published by our research group and showed a total of 17 (6.6%) aseptic revisions, with a 1-year survival of 87.4% (SE 3.8%) using the Kaplan-Meier method [10]. Another study showed a 2-year survival of 92% for the same acetabular cup with another ceramic bearing [11]. Mid-term analysis of the same implant showed an aseptic loosening rate of 10% after 48.6 months [12]. Since CoC seems to be a good option to overcome wear and late revisions, the reason for early failure of this type of implant is important to clarify. Although different theories were discussed in the three aforementioned studies, to our knowledge, no other study published long-term results after the osseointegration phase of three years [10–12].

The aim of this study is to investigate the ten year outcomes of this acetabular component and to differentiate between potential causes and identify risk factors for aseptic loosening. These outcomes can be helpful to contribute to the search of the perfect implant.

Our hypothesis was that the TH+ acetabular component would stabilize over time after a period of high early failure rate.

## Materials and methods

### Ethical approval

Ethical approval of this retrospective cohort study was given by the local ethics committee review board. The design and reporting were performed in accordance to the Strengthening the Reporting of OBservational studies in Epidemiology (STROBE) statement. This research was conducted in regard of the Declaration of Helsinki.

### Study design and setting

We retrospectively collected the ten year follow-up outcomes of our prospectively documented series elective total hip arthroplasty procedures with a seleXys TH+ cup performed between January 2009 and October 2010. The short-term outcomes of this study were published earlier by our research group with a smaller cohort since not all patients had reached a meaningful minimal follow-up term [10]. All prospectively documented data were checked for correctness and complemented if necessary. The retrospective screening of patient records after ten years of follow-up was performed by a researcher (XXX) that was not involved in the surgical process. When no additional information was available, patients were considered to be lost to follow-up. The last date of follow-up at the hospital, date of death or date of cup revision was used to calculate the follow-up time. All outcomes were checked by a second researcher (XXX), also not involved in the surgical process.

### Eligibility

All indications for THA included in this study were primary osteoarthritis (OA), secondary OA due to prior osteotomy, prior osteosynthesis or failure of conservative treatment of a hip fracture, rheumatoid arthritis, avascular necrosis or congenital dysplasia of the hip, and femoral fractures close to the joint. Indications were categorized as primary versus secondary OA or primary traumatic treatment. If initial cup stability was not achieved and additional screw fixation was needed, patients were excluded from this study.

### Surgical procedure and product information

All procedures were performed at Tergooi using an anterolateral approach under standard antibiotic prophylaxis consisting of 2-grams cefazoline pre-operatively and two doses of 2-grams post-operatively. All THA procedures were performed by three experienced orthopaedic surgeons or under their direct supervision. The preparation of the acetabulum and femur was according to the surgical technique described by the manufacturer of the implants. After implantation of the seleXys TH+ cup (Mathys Medical), a Ceramys (Mathys Medical) ceramic insert of aluminia-thoughened zirconia (ATZ) was used in all cases. We used the Mathys CBH stem or Mathys offset stem, which is a forged rough-blasted surface stem made of a titanium-aluminum-niobium alloy. If a longer stem was needed, we used a 20% longer Zimmer Alloclasic Zweymuller revision stem (Zimmer GmbH, Winterthur, Switzerland). Neck length was available in four different sizes to gain optimal stability of the whole implant. The aimed femoral offset and leg length were measured accordingly to be identical to the contralateral side. Ceramic heads of 32 mm were used in cups up to 50 mm and 36-mm heads for cups of 52 mm and larger, both with matching inlays. After surgery, standard post-operative rehabilitation under supervision of a physical therapist consisted of immediate full weight bearing with crutches for six weeks. Patients were assessed in a standard care follow-up protocol with X-rays at six, 12, 26, and 52 weeks post-surgery and yearly afterwards.

### Outcomes

Patient demographics and implant information were recorded at baseline, including age, gender, indication for THA (primary or secondary OA or primary traumatic treatment), duration of surgery, cup size, head size, stem size, and complications during surgery and during post-operative follow-up.

The primary outcome was cup revision due to aseptic loosening. Progressive radiolucency with pain during weight bearing or clear displacement of more than 3–5 mm and inclination more than 3°–5° was defined as loosening [13–15]. If purulent discretion, positive cultures peri-operatively, or high suspicion due to high infection parameters (CRP or leukocytes) were seen, cases were defined as septic loosening.

Secondary outcomes were component revision, stated as a procedure by which the cup, the stem, or both were revised and re-operation for any reason. Additionally, potential predictive factors for revision due to aseptic loosening were evaluated.

### Statistical analysis

Statistical analyses were performed with Statistical Package for Social Sciences (SPSS) version 26.0 (SPSS Inc. Chicago, IL). Distribution of continuous variables was assessed using the Shapiro-Wilk tests. Normally distributed variables are stated as medians with interquartile ranges (IQRs). Categorical data are described as numbers with accompanying proportions. Since follow-up was long and the population relatively old, both Kaplan-Meier (KM) and competing risk (CR) analyses (with death as competing risk) were performed to determine the survival of the cup. Survival of the cup was expressed as cumulative revision rates and cumulative revision incidence, respectively. The association between potential predictive factors and cup revision was assessed by use of univariate Cox regression analyses and expressed as hazard ratio (HR) with 95% confidence intervals (CIs). Statistical significance was considered if *p* values were less than 0.05.

## Results

A total of 315 elective total hip procedures in 307 patients were performed. Table 1 shows the baseline characteristics of the 307 patients and operative information of the 315 elective total hip procedures performed on these patients. Peri-operative complications occurred in seven cases (2.2%) with five fractures of the greater trochanter (1.6%), one fissure around the stem treated conservatively (0.3%), and one fausse route (0.3%) which was operated again the day after. Complications related to the surgical site were post-operative bleeding (0.3%), haematoma (0.6%), and persistent wound leakage (1.3%). Two patients (0.6%) died respectively 22 and 30 days after surgery after post-operative organ failure, due to deterioration of congestive heart failure in one case and acute kidney failure in the other patient. No ceramic liner fracture was observed in our study. Hip dislocation occurred in three cases (1.0%).

**Table 1:** Baseline characteristics and operative information of the 315 elective total hip procedures performed on 307 THA patients

Characteristic	Outcome
Gender, *n* (%)	
• Female • Male	216 (68.6) 99 (31.4)
Age at operation in years, median [IQR]	71 [64; 77]
Indication, *n* (%)	
• Primary OA • Secondary OA • Primary traumatic treatment	274 (86.9) 37 (11.7) 4 (1.3)
Operation time in minutes, median [IQR]	55 [43; 69]
Cup size in mm, median [IQR]	52 [52; 54]
Head size in mm, *n* (%)	
• 32 • 36	77 (24.4) 238 (75.6)
Stem size, median [IQR]	5 [4; 6]
Stem type, *n* (%)	
• Mathys CBS • Mathys CBS Offset • Alloclassic Zweymuller revision stem	297 (94.3) 14 (4.4) 4 (1.3)
Neck length, *n* (%)	
• Small • Medium • Large • Extra-large	129 (41.0) 120 (38.1) 65 (20.6) 1 (0.3)

### Primary outcome

**Fig. 1:**
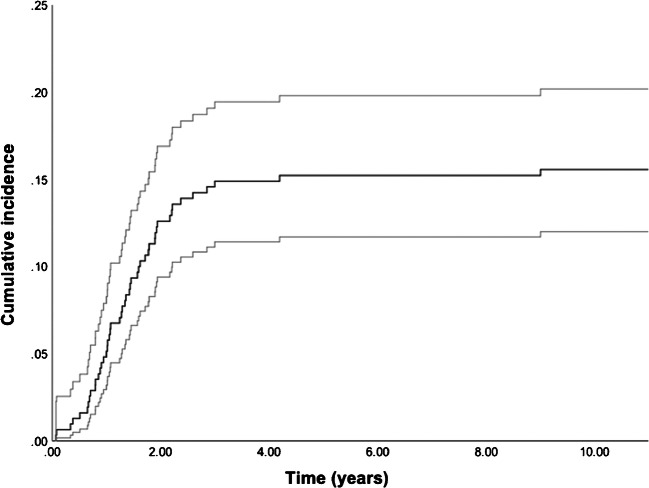
Cumulative incidence of cup revision over time, with upper and lower limits of 95% CI

Competing risk analysis demonstrated a 10-year cumulative incidence of cup revision, due to aseptic loosening, of 15.6% (95% CI 12.0–20.2). A total of 12 cases (3.8%) were lost to follow-up, and 57 died during follow-up (18.1%) (Fig. 1).

**Fig. 2:**
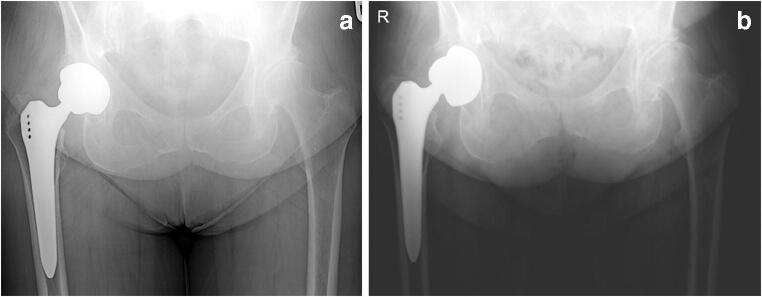
Example of a case with aseptic loosening. **a** Direct post-operative X-ray. **b** X-ray at 9 months follow-up with clear loosening of the cup

With a median follow-up of 9.7 years [IQR 4.4; 10.3], a total of 48 TH+ (15.2%) were revised due to aseptic loosening. In five cases (1.6%), the stem was revised due to aseptic loosening as well. A total of 57 (18.1%) patients had died. Follow-up time ranged from one month up to 11 years. The median time point of cup revision was 15.8 months [IQR 10.3; 22.9]. Respectively, 81.3% (*n* = 39) and 95.8% (*n* = 46) of all cup revisions for aseptic loosening were performed within the first two and three years. One cup was revised after 50 months following ongoing complaints two years post-surgery. A bone scintigraphy performed just before revision confirmed aseptic loosening. The second late revision was performed 9.0 years post-surgery. This patient presented with complaints three years earlier showing migration of the cup on X-ray. Revision was postponed due to mild complaints in preference of the patient. All retrieved cups showed a lack of bony ingrowth on the implant. Figure 2 displays an example of aseptic loosening in our study

### Secondary outcomes

Cumulative revision rates at ten year follow-up are shown in Table 2. Component revision was performed in 56 (18.8%) patients. A total of 45 (14.2%) cup revisions were performed of which 43 (13.6%) due to aseptic loosening and two cases (0.6%) due to infection. Three stem revisions (1.0%) were performed due to aseptic loosening. Both the stem and cup were revised in eight cases (2.6%) with five cases (1.6%) due to aseptic loosening and three resection arthroplasties according to Girdlestone (1.0%) due to infection.

**Table 2:** Cumulative revision rates in % (95% CI) after 10-year follow-up for all endpoints; using Kaplan-Meier analysis

Endpoint at 10-year follow-up	Number of events	Cumulative 10-year revision rate in % (with 95%CI)
Cup revision (aseptic loosening)	48	16.1% (12.0-20.2)
Component revision	56	18.8% (14.3-23.3)
Reoperation	62	20.8% (16.1-20.8)

In 62 cases (20.8%), any re-operation was performed. In addition to the 56 component revisions, five periprosthetic fractures (1.6%) needed re-operation, and one exploration without intervention (0.3%) was performed due to complaints of inexplicable pain.

Univariate Cox regression analyses for determining predictive factors for revision due to aseptic loosening showed no significant outcomes as presented in Table 3.

**Table 3:** Hazard ratios (HRs) for potential predictive factors for cup revision due to aseptic loosening (with 95% CI)

Predictive factor	Hazard ratio (with 95%CI)	*p* value
Male gender	0.87 (0.46–1.64)	0.66
Age	1.24 (0.69-2.22)	0.48
Primary vs. secondary	2.07 (0.64–6.67)	0.22
Cup size	1.02 (0.92–1.13)	0.71
Stem size	0.88 (0.74–1.05)	0.15
Head size (36 vs. 32)	0.91 (0.48–1.71)	0.76

## Discussion

The main finding of this 10-year follow-up retrospective cohort study of 315 THA with the seleXys TH+ acetabular cup (Mathys Medical) with a ceramic-on-ceramic bearing is an unacceptable high cumulative revision incidence (15.2%) due to aseptic loosening, which stabilizes over time after a period of high early failure. Although this cup is withdrawn from the market, the reason for failure still remains unclear. This outcome confirms our hypothesis and is consistent with a same trend in literature, where revision rates of 8% after two year follow-up and 10% after 48.6 months are shown [11, 12]. Revision due to aseptic loosening was seen after a median of 1.32 years [0.86–1.90] with 96% revised within three year follow-up. Two additional cases showed complaints and signs of aseptic loosening long before revision. Our main outcome is less likely to be due to a problem with the primary stability since the initial reaming and press-fit feeling during surgery were satisfactory and comparable with other designs. This indicates a problem with subsequent transition from primary to definitive stability by osseointegration.

**Fig. 3:**
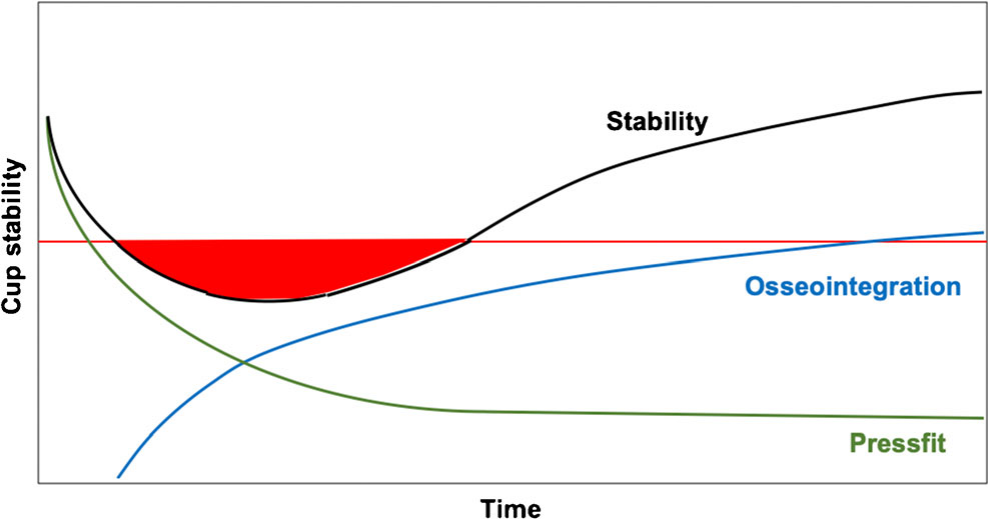
Distribution of primary and secondary stability over time. The minimum stability needed for safe fixation is indicated with the red line. In the red area, the cup is at risk for loosening if transition from press fit to definitive stability by osseointegration is jeopardized

Our theory is that after implantation of the cup, primary stability, mainly obtained by press-fit, decreases over time. Subsequent transition to secondary stability is achieved by an increase in osseointegration, which is influenced by several factors. These processes can initially result in a decrease of the overall stability of the cup, which can bring the implant at risk for loosening if osseointegration is threatened. An increase of overall stability to the definitive stability of the implant is obtained when osseointegration becomes sufficient. This theory was stated before by our research group and is visualized in Fig. 3 [10]. Several studies in literature could confirm this theory using radiostereometric analysis (RSA) measuring migration by translation and rotation, which is observed mostly in the first six months post-operatively and stabilizes in two to three years post-operatively by osseointegration [16–20]. These results are supported by studies measuring periacetabular bone mineral density (BMD), which changes during osseointegration, showing loss of BMD in the first six months after surgery and restores to baseline in at least two years [21–23]. Brodt et al. stated that a limitation of failure of the TH+ to the period of osseointegration could be ruled out [12]. Since the period of RSA migration and change of BMD is covering the majority of aseptic loosening in our study, it refutes the statement of Brodt et al. and funds our theory of a problem with osseointegration [12].

**Fig. 4:**
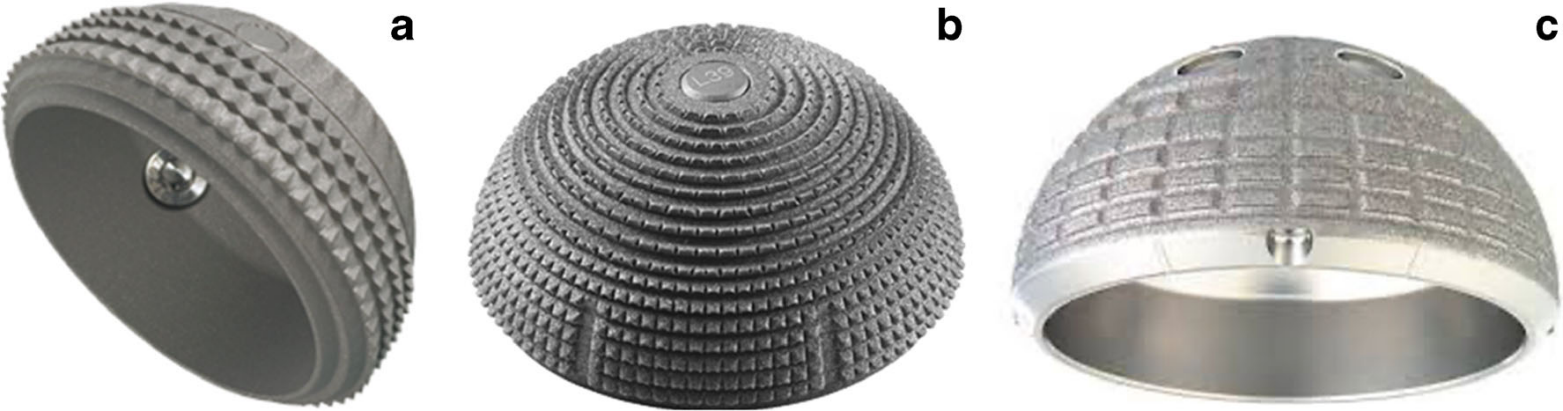
Macroscopic cup design of the **a** SeleXys TH+ (Mathys Medical), **b** Allofit (Zimmer), **c** Cerafit-R (Ceraver)

Osseointegration can be threatened by several factors. For example, implant design, by the biocompatibility, microscopic structure, and macroscopic design of the cup. The seleXys TH+ cup has a titanium alloy, which has good biocompatibility with bone [24]. The microscopic texture is a corundum blasted roughened surface which has a highly osteoconductive nature [25]. Furthermore, the macroscopic cup design has a greater influence on stability than surface modification if a rough surface is chosen [26]. The macroscopic cup design of the TH+ has tetrahedrons with a height of 0.65 ± 0.1 mm on the peripheral ring, as shown in Fig. 4. Literature has shown that macroscopic spikes in this area decrease primary stability and since the load on the implant is transferred to this acetabular rim, the TH+ becomes more vulnerable to loosening [27, 28]. The Allofit cup (Zimmer) has the same cup design, except for comparable shaped smaller teeth of 0.4–0.6 mm height on the whole surface of the cup, as shown in Fig. 4. This cup shows an 11-year survival rate of 98% with only one aseptic acetabular loosening [29].

Another specific feature of the TH+ design is that the rim protrudes 4 mm from the acetabulum. This could lead to impingement between the cup and neck and can provoke loosening during transition from primary to secondary stability. A large cohort study of different retrieved cup designs showed that rim impingement occurs in 56% of the implants with a higher occurrence among components with an elevated rim, making it unlikely that rim impingement would not occur in the TH+ [30]. Brodt et al. compared the TH+ with a control group with a Cerafit-R cup (Ceraver, Roissy, France), which had the same lateral overlap of 4 mm without a high rate of aseptic loosening, as visualized in Fig. 4 [12]. Since the impingement force gets transferred to the bone-implant interface through the bearing and only leads to aseptic loosening in the TH+ cup and not in comparable cup designs, it becomes more likely that one of the reasons of failure are the characteristic bigger teeth of tetrahedrons only located at the peripheral rim.

The bearing can influence transition to definitive stability by its friction and stiffness. CoC bearings have the lowest friction between the head and cup compared with all other articulations in several biomechanical studies, excluding this as a potential reason for bone-implant interface failure [31, 32]. The total stiffness of the implant can be raised by a ceramic bearing. As a result, the forces of normal weight bearing and rim impingement get less absorbed by the coupling and implant than in CoPE and get transferred to the bone-implant interface. This causes shear forces which jeopardize the initial press-fit and hamper osseointegration. Several studies mentioned that hard bearings like CoC might have an influence on the transition to definitive stability by osseointegration, but there is still a lack of evidence [33–35]. Biomechanical analysis of several implants showed that the combination of the TH+ with a Ceramys inlay is the stiffest [36]. Ilchmann et al. showed an 8% revision rate at two years of the TH+ with a much lower stiffness Bionit ceramic inlay, and this inlay had good mid-term results with different cups with a revision rate of 1.4% and 1.0%; thus, the loosening in the study of Ilchmann will probably be due to the cup [37, 38]. Our higher two year revision rate of 12.4% indicates that the stiffness can be a reason to explain the difference in survival. However, the study of Brodt et al. showed that half of their revisions were a TH+ with a polyethylene liner. RSA showed that another CoC bearing compared with CoPE had no difference in migration after two years [39]. Since these results were only seen in the aforementioned two studies with a small number of patients, their outcomes support that the problem of the high aseptic loosening rate of the TH+ is multifactorial and the stiff ceramic bearing is one of the main reasons.

Other factors than the implant or bearing are surgical technique, the status of the implant bed bone quality, undisturbed healing phase, loading conditions, and patient-specific conditions like age, comorbidity, medication, or intoxications [40]. Patient-related factors showed no differences in our short-term follow-up study when compared with an equally matched group with another cup with CoC bearing. In this study, the same patients were included and the same experienced surgeons used the same approach with the same rehabilitation program for all patients. Even though patient-related factors were not the main focus of this study, and more power might be needed to show significant differences, these outcomes indicate that it is more likely that the implant and its bearing are the reason for aseptic loosening.

Focusing on the long-term survival rate of our ten year study of CoC bearing in THA, a revision rate of 18.8% was observed, resulting in a survival rate of 82.2%. In literature, divergent survival rates of CoC on long-term are observed, with a 15-year follow-up study showing a survival rate of 92%, whereas another 20-year follow-up study showed a survival rate of 99.7% [41, 42]. Our higher survival rates can be explained by the fact that our cohort showed an extremely high early revision rate, since stabilization of revision procedures was observed, with 95.8% (*n* = 46) of the revisions performed in the first three years.

Based on the outcomes of this study complemented with available literature, we believe that the macroscopic cup design with big tetrahedrons only at the peripheral rim together with stiff Ceramys bearing of the TH+ could be the main reasons for aseptic loosening. These factors can make the implant vulnerable for loosening due to shear forces on the bone-implant interface in combination with loss of stability and may interfere with the process of osseointegration. This can reduce bony ingrowth and thus long-term stability, causing migration and aseptic loosening on both the short and long term [43]. Larger cohorts or RSA studies are needed to confirm the role of bearings on primary stability, osseointegration, and revision in THA.

## Conclusion

The 10-year results of seleXys TH+ cup with Ceramys CoC bearing showed an unacceptable high aseptic loosening rate, which stabilized over time after a high early failure incidence. This could be attributed to a problem with osseointegration during the transition of primary to definitive stability.

## Data Availability

Not available for further research

## References

[1] Le Cann S, Galland A, Parratte S, Rosa B, Argenson JN, Chabrand P (2012). Biomechanical testing of the primary stability of macro and micro-roughnesses acetabular cups: a numerical and an experimental study. Comput Method Biomec.

[2] Michel A, Bosc R, Meningaud JP, Hernigou P, Haiat G (2016). Assessing the acetabular cup implant primary stability by impact analyses: a cadaveric study. PLoS One.

[3] Perona PG, Lawrence J, Paprosky WG, Patwardhan AG, Sartori M (1992). Acetabular micromotion as a measure of initial implant stability in primary hip arthroplasty. an in vitro comparison of different methods of initial acetabular component fixation. J Arthroplast.

[4] Mavrogenis AF, Dimitriou R, Parvizi J, Babis GC (2009). Biology of implant osseointegration. J Musculoskel Neuron.

[5] Pilliar RM, Lee JM, Maniatopoulos C (1986) Observations on the effect of movement on bone ingrowth into porous-surfaced implants. Clin Orthop Relat R. (208):108-113 3720113

[6] Melvin JS, Karthikeyan T, Cope R, Fehring TK (2014). Early failures in total hip arthroplasty -- a changing paradigm. J Arthroplast.

[7] Dumbleton JH, Manley MT, Edidin AA (2002). A literature review of the association between wear rate and osteolysis in total hip arthroplasty. J Arthroplast.

[8] McCalden RW, MacDonald SJ, Rorabeck CH, Bourne RB, Chess DG, Charron KD (2009). Wear rate of highly cross-linked polyethylene in total hip arthroplasty. A randomized controlled trial. J Bone Joint Surg Am.

[9] Hernigou P, Zilber S, Filippini P, Poignard A (2009). Ceramic-ceramic bearing decreases osteolysis: a 20-year study versus ceramic-polyethylene on the contralateral hip. Clin Orthop Relat R.

[10] Haverkamp D, Westerbos S, Campo MM, Boonstra RH, Rob Albers GH, van der Vis HM (2013). Early loosening of a press-fit cup with ceramic-on-ceramic articulation: our early results. Arch Orthop Traum Su.

[11] Ilchmann T, Zwicky L, Gersbach S, Clauss M (2014). Poor outcome of a spherical pressfit cup with a modern ceramic liner: a prospective cohort study of 181 cups. Hip Int.

[12] Brodt S, Matziolis G, Windisch C, Gosse A, Spalteholz M, Gahr RH (2015). High failure rate of a new pressfit cup in mid-term follow-up. Int Orthop.

[13] Malchau H, Karrholm J, Wang YX, Herberts P (1995). Accuracy of migration analysis in hip arthroplasty. Digitized and conventional radiography, compared to radiostereometry in 51 patients. Acta Orthop Scand.

[14] Massin P, Schmidt L, Engh CA (1989). Evaluation of cementless acetabular component migration. An experimental study. J Arthroplast.

[15] Udomkiat P, Wan Z, Dorr LD (2001). Comparison of preoperative radiographs and intraoperative findings of fixation of hemispheric porous-coated sockets. J Bone Joint Surg Am.

[16] Grosser D, Benveniste S, Bramwell D, Krishnan J (2013). Early migration of the R3 uncemented acetabular component: a prospective 2 year radiostereometric analysis. J Surg.

[17] Klerken T, Mohaddes M, Nemes S, Karrholm J (2015). High early migration of the revised acetabular component is a predictor of late cup loosening: 312 cup revisions followed with radiostereometric analysis for 2-20 years. Hip Int : the journal of clinical and experimental research on hip pathology and therapy.

[18] Laende EK, Richardson CG, Dunbar MJ (2020). Migration and wear of a dual mobility acetabular construct at 3 years measured by radiostereometric analysis. J Arthroplast.

[19] Nieuwenhuijse MJ, Valstar ER, Kaptein BL, Nelissen RG (2012). Good diagnostic performance of early migration as a predictor of late aseptic loosening of acetabular cups: results from ten years of follow-up with Roentgen stereophotogrammetric analysis (RSA). J Bone Joint Surg Am.

[20] Rohrl SM, Li MG, Nilsson KG, Nivbrant B (2007). Very low wear of non-remelted highly cross-linked polyethylene cups: an RSA study lasting up to 6 years. Acta Orthop.

[21] Gerhardt DM, Smolders JM, Roovers EA, Rijnders TA, van Susante JL (2019). Changes in periacetabular bone mineral density five years after resurfacing hip arthroplasty versus conventional total hip arthroplasty. Hip Int.

[22] Salemyr M, Muren O, Eisler T, Boden H, Chammout G, Stark A, Skoldenberg O (2015). Porous titanium construct cup compared to porous coated titanium cup in total hip arthroplasty. A randomised controlled trial. Int Orthop.

[23] Venesmaa PK, Kroger HP, Jurvelin JS, Miettinen HJ, Suomalainen OT, Alhava EM (2003). Periprosthetic bone loss after cemented total hip arthroplasty: a prospective 5-year dual energy radiographic absorptiometry study of 15 patients. Acta Orthop Scand.

[24] Hu CY, Yoon T-R (2018). Recent updates for biomaterials used in total hip arthroplasty. Biomater Res.

[25] Hacking SA, Bobyn JD, Tanzer M, Krygier JJ (1999) The osseous response to corundum blasted implant surfaces in a canine hip model. Clin Orthop Relat R. (364):240-253. 10.1097/00003086-199907000-00031 10.1097/00003086-199907000-00031 10416415

[26] Schreiner U, Simnacher M, Scheller G, Scharf H (2007). The influence of different surface treatments on the primary stability of cementless acetabular cups: an in vitro study. Biomed Eng.

[27] Le Cann S, Galland A, Rosa B, Le Corroller T, Pithioux M, Argenson JN, Chabrand P, Parratte S (2014). Does surface roughness influence the primary stability of acetabular cups? A numerical and experimental biomechanical evaluation. Med Eng Phys.

[28] Schmidt R, Kress AM, Nowak M, Forst R, Nowak TE, Mueller LA (2012). Periacetabular cortical and cancellous bone mineral density loss after press-fit cup fixation: a prospective 7-year follow-up. J Arthroplast.

[29] Streit MR, Weiss S, Andreas F, Bruckner T, Walker T, Kretzer JP, Ewerbeck V, Merle C (2014). 10-year results of the uncemented Allofit press-fit cup in young patients. Acta Orthop.

[30] Shon WY, Baldini T, Peterson MG, Wright TM, Salvati EA (2005). Impingement in total hip arthroplasty a study of retrieved acetabular components. J Arthroplast.

[31] Brockett C, Williams S, Jin Z, Isaac G, Fisher J (2007). Friction of total hip replacements with different bearings and loading conditions. J Biomed Mater Res B.

[32] Vrbka M, Necas D, Bartosik J, Hartl M, Krupka I, Galandakova A, Gallo J (2015). Determination of a friction coefficient for THA bearing couples. Acta Chir Orthop Tr.

[33] Aldrian S, Nau T, Gillesberger F, Petras N, Ehall R (2009). Medium-term analysis of modern ceramic-on-ceramic bearing in THA. Hip Int.

[34] Bottner F, Su E, Nestor B, Azzis B, Sculco TP, Bostrom M (2005). Radiostereometric analysis: the hip. HSS Jrnl.

[35] Floerkemeier T, Schwarze M, Hurschler C, Gronewold J, Windhagen H, von Lewinski G, Budde S (2017) The influence of tribological pairings and other factors on migration patterns of short stems in total hip arthroplasty. Biomed Res Int. 8756432-8756432. 10.1155/2017/8756432 10.1155/2017/8756432 PMC5406728 28497067

[36] Hothan A, Huber G, Weiss C, Hoffmann N, Morlock M (2011). Deformation characteristics and eigenfrequencies of press-fit acetabular cups. Clin Biomech (Bristol, Avon).

[37] Chevillotte C, Pibarot V, Carret JP, Bejui-Hugues J, Guyen O (2011). Nine years follow-up of 100 ceramic-on-ceramic total hip arthroplasty. Int Orthop.

[38] Choy W-S, Kim KJ, Lee SK, Bae KW, Hwang YS, Park CK (2013). Ceramic-on-ceramic total hip arthroplasty: minimum of six-year follow-up study. Clin Orthop Surg.

[39] Zhou ZK, Li MG, Borlin N, Wood DJ, Nivbrant B (2006). No increased migration in cups with ceramic-on-ceramic bearing: an RSA study. Clin Orthop Relat R.

[40] Apostu D, Lucaciu O, Berce C, Lucaciu D, Cosma D (2018). Current methods of preventing aseptic loosening and improving osseointegration of titanium implants in cementless total hip arthroplasty: a review. J Int Med Res.

[41] Pitto R. (2018) Ceramic-on-ceramic total hip arthroplasty: long-term results in a national registry. J Bone Joint Surg. 100-B: 5-5. 10.1302/1358-992X.2018.5.005.

[42] Pijls BG, Nieuwenhuijse MJ, Fiocco M, Plevier JW, Middeldorp S, Nelissen RG, Valstar ER (2012). Early proximal migration of cups is associated with late revision in THA: a systematic review and meta-analysis of 26 RSA studies and 49 survivalstudies. Acta Orthop.

[43] Kim YH, Park JW, Kim JS (2016). Long-term results of third-generation ceramic-on-ceramic bearing cementless total hip arthroplasty in young patients. J Arthroplast.

